# New Developments in Biomarkers for Atopic Dermatitis

**DOI:** 10.3390/jcm4030479

**Published:** 2015-03-16

**Authors:** Judith L. Thijs, Wouter van Seggelen, Carla Bruijnzeel-Koomen, Marjolein de Bruin-Weller, DirkJan Hijnen

**Affiliations:** Department of Dermatology and Allergology, University Medical Center Utrecht, Heidelberglaan 100, 3584 CX Utrecht, The Netherlands; E-Mails: j.thijs@umcutrecht.nl (J.L.T.); wouterv.seggelen@gmail.com (W.S.); c.bruijnzeel@umcutrecht.nl (C.B.-K.); m.s.debruin-weller@umcutrecht.nl (M.B.-W.)

**Keywords:** atopic dermatitis, eczema, biomarkers, disease severity, stratification, dried blood spots, saliva

## Abstract

The application of biomarkers in medicine is evolving. Biomarkers do not only give us a better understanding of pathogenesis, but also increase treatment efficacy and safety, further enabling more precise clinical care. This paper focuses on the current use of biomarkers in atopic dermatitis, new developments and future perspectives. Biomarkers can be used for many different purposes, including the objective determination of disease severity, confirmation of clinical diagnosis, and to predict response to treatment. In atopic dermatitis, many biomarkers have been investigated as a marker for disease severity. Currently serum thymus and activation-regulated chemokine (TARC) is the superior biomarker for assessing disease severity. However, we have recently shown that the use of a panel of serum biomarkers is more suitable for assessing disease severity than an individual biomarker. In this overview, we will discuss alternative sources for biomarkers, such as saliva and capillary blood, which can increase the user friendliness of biomarkers in atopic dermatitis (AD). Both methods offer simple, non-invasive and cost effective alternatives to venous blood. This provides great translational and clinical potential. Biomarkers will play an increasingly important role in AD research and personalized medicine. The use of biomarkers will enhance the efficacy of AD treatment by facilitating the individualization of therapy targeting the patients’ specific biological signature and also by providing tools for predicting and monitoring of therapeutic response.

## 1. Introduction

The World Health Organization has defined a biomarker as “any substance, structure, or process that can be measured in the body or its products and influence or predict the incidence of outcome or disease” [1]. Biomarkers have been used in clinical practice for many years. For example, prostate-specific antigen (PSA) is a commonly used biomarker and is used for the follow-up in prostate cancer, as well as serum creatinine levels for diagnosing renal insufficiency [2,3]. Additionally, in the past decade rapid advances in genomic and proteomic technologies have generated a plethora of candidate biomarkers ranging from antibodies, microbes, DNA, RNA, lipids, metabolites to proteins [4].

The application of biomarkers in medicine is evolving. Biomarkers do not only give us a better understanding of pathogenesis, but also increase treatment efficacy and safety further enabling more precise clinical care.

Biomarkers can be categorized in different types depending on their specific characteristics. They can be used to identify the risk of developing a disease (screening biomarkers), to identify a disease (diagnostic biomarker), predict disease progression (prognostic biomarker), mark a particular pharmacological response (pharmacodynamic biomarkers), and for monitoring disease activity and clinical response to an intervention or as a surrogate endpoint in clinical trials (severity biomarker) [5]. Moreover, biomarker based stratification may identify clinically relevant subgroups and help to provide effective targeted therapies (predictive biomarker) [6].

This paper focuses on the current use of biomarkers in atopic dermatitis (AD), new developments and future perspectives.

## 2. Biomarkers in Atopic Dermatitis

### 2.1. Diagnostic Biomarker

Atopic dermatitis remains a clinical diagnosis without an objective and reliable biomarker to confirm the diagnosis. Classically AD has been divided into two subtypes, intrinsic *versus* extrinsic. Patients with an intrinsic form show normal total IgE levels, without specific IgE and have also been termed non-atopic or non-allergic dermatitis. Comparatively, patients with an extrinsic form of AD show high total IgE levels and are often sensitized to multiple allergens [7]. Since total serum IgE levels are not increased in about 20% of AD patients, total serum IgE cannot be used as a diagnostic biomarker for all AD patients [7]. Moreover, total serum IgE levels are also increased in patients with other atopic diseases such as allergic rhinoconjunctivitis and allergic asthma [8]. Using a genomic profiling approach, Suárez-Farinas *et al.*, recently showed common disease-defining features in patients with intrinsic and extrinsic AD [9]. Interestingly, they found similar Th2 type immune activation in intrinsic and extrinsic AD patients, suggesting that Th2 is not the only cause of high IgE levels in patients with extrinsic AD.

### 2.2. Severity Biomarker

Patients with severe disease tend to have higher IgE levels, but there are also patients with severe eczema that do not show increased IgE levels. Total serum IgE levels are therefore not a reliable biomarker for disease severity [10]. Other frequently reported serum biomarkers for disease severity in AD include eosinophilic cationic protein (ECP) [11], IL-2R [12], and thymus and activation-regulated chemokine (TARC/CCL17) [13]. In a systemic review on serum biomarkers for disease severity in AD we found that serum TARC levels showed the best correlation to disease severity, with weighted mean R-values of 0.51 and 0.63 in longitudinal and cross-sectional studies, respectively [14]. However, we have shown that patients with severe AD can have serum TARC levels in the normal range, and on the other hand patients with mild to moderate disease may express high TARC levels [15]. This might be explained by the large number of biological pathways involved in the pathogenesis of AD and the clinical heterogeneity.

Thus, we hypothesized that a combination of biomarkers can overcome these problems, by providing more information on different biological pathways and would be applicable to different phenotypical subtypes and thereby correlate better to disease severity. We recently demonstrated that indeed a multivariate signature including four serum biomarkers showed a correlation coefficient of 0.86 to disease severity measured by the six area, six sign atopic dermatitis severity score (SASSAD) [16]. Although this was only a pilot study in 17 patients, it showed that using a panel of biomarkers may be necessary in a multifactorial, complex disease such as AD [17].

Currently, there is no gold standard for measurement of disease severity in AD and more than 20 different composite indices have been described [18]. The Harmonizing Outcome Measurements in Eczema (HOME) initiative is working on a core set of outcome measures but currently there is no consensus (www.homeforeczema.org). The ultimate goal from the perspective of evidence-based medicine is to achieve worldwide consensus to consistently apply a single valid, reliable, and feasible instrument to measure disease severity for AD. We suggest that the use of a panel of biomarkers can add important information or even substitute clinical endpoints, and can be used in daily practice to track changes in disease activity and adapt treatment accordingly. Moreover, the use of biomarkers as a surrogate endpoint in clinical trials will improve comparisons across trials and facilitate meta analyses. With the recent introduction of the first biological for AD [19] further studies are needed to explore the use of a panel of biomarkers as a disease severity measure and possibly treatment predictive tool.

### 2.3. Predictive Biomarker

The introduction of novel agents and “targeted” therapies also drives the need for predictive biomarkers. Because of disease heterogeneity, stratification of subgroups is essential in the development of such a predictive tool. Biomarkers can be used to identify subgroups of patients with shared “biological” characteristics, which are more likely to respond favorably to a given therapy. Using stratification, targeted therapies can be assigned to different subgroups, making personalized medicine possible.

## 3. Alternative Ways to Measure Biomarkers

Blood is the most commonly used body fluid for biomarker measurements [20]. However, collection of blood is invasive and less suitable for use in the field because of the need for trained personnel. It is also less favored in pediatric medicine, especially since atopic dermatitis usually presents in childhood. The same is true for gene expression profiles determined in skin biopsies. Studies have identified specific molecular signatures for AD patients [21], and changing expression profiles during therapy [19,22]. Although tissue biomarker expression patterns may also be extremely helpful in stratification and the identification of new pathways involved in the pathogenesis of AD, they require trained personnel and specialized labs.

We therefore explored alternatives for use in daily practice and longitudinal studies. Specifically dried blood spots (DBS) and saliva as potential alternatives.

DBS have been used for decades in screening for inherited metabolic diseases in newborns [23] and can be obtained using a simple, minimally invasive, nearly painless procedure that can be done by the patients themselves.

Secondly, saliva is a mirror of the body’s health as a wide spectrum of biomolecules is transported from the blood capillaries through the epithelium of salivary glands [24]. Salivary cortisol levels are for instance routinely used as a biomarker of psychological stress. We have preliminary data showing that several inflammatory biomarkers can be measured in saliva samples from AD patients. DBS and saliva may be used as an accurate non-invasive alternative to serum measurements; both methods will subsequently be discussed.

### 3.1. Dried Blood Spots (DBS)

DBS are samples from drops of capillary whole blood collected from a finger stick and dried on filter paper [25]. Filter paper was first used as a scientific tool in 1815 by the Swedish chemist Jöns Berzelius. Robert Guthrie is widely credited as being the first to use blood dried on filter paper (so-called Guthrie cards) to diagnose phenylketonuria in neonates in 1963 [26]. Since that time, filter paper has become a commonly used method of storing and transporting diverse specimens. Different aspects of the use of DBSs have been reviewed; DBS used for newborn screening assays, for epidemiological studies, human immunodeficiency virus (HIV) detection and monitoring, virology and drug assays [27,28].

Collection of a DBS is a relatively simple and minimally invasive, nearly painless procedure. The participant’s finger is first wiped with alcohol. A sterile lancet is then used to puncture the skin. The first drop of blood is dabbed, the subsequent four drops of blood are applied to filter paper. The paper is left to dry for a few hours and then stored at room temperature or refrigerated before shipment to the laboratory [25,26]. Most analytes remain stable for more than a week in filter paper when stored at room temperature [29].

Sample processing is relatively easy and the requirements are minimal. To extract the analytes from DBS, small discs are punched from the filter paper and eluted with a buffer. Current immunological essay technologies, like multiplex bead-based immunoassay and mass spectrometry, require only small volumes, which provides the ability to measure multiple proteins and peptides from a single DBS sample [26]. Chambers *et al.* demonstrated that a panel of 40 proteins could be quantitatively extracted from DBS using highly-multiplexed mass spectrometry [30].

The ease of sample collection, minimal training requirements, and self-applicability by the patient at home are great advantages of DBS sampling. The minimal volume requirements, the stability of analytes for months to years and the ease of sample processing offers practical and financial advantages, making DBS also very suitable for storage in biobanks [31]. Additionally, the low burden and minimally invasive procedure is better suited for pediatric studies and also allows collection of multiple samples over time in longitudinal study designs [31].

### 3.2. Saliva

In humans, three major glands produce saliva: the parotid, submandibular and sublingual gland. Together they produce over 90% of the total amount of saliva. Saliva is also produced by hundreds of small salivary glands, spread throughout the oral cavity [32]. Epithelial cells generate saliva and it is secreted via salivary ducts into the oral cavity.

Salivary glands are highly permeable and are surrounded by capillaries. These characteristics enable free exchange of molecules from blood to the acinus and therefore it is thought that biomarkers, circulating in blood, can diffuse from blood to the acinar cells and eventually secreted in saliva [33]. This makes saliva an interesting source for the measurement of biomarkers using epigenetic, transcriptomic, proteomic and metabolomic approaches [34]. Over the last decade, numerous studies have investigated biomarkers in saliva related to specific diseases. This includes studies on Sjögren’s syndrome, rheumatic-, cardiovascular-, and periodontal diseases [33,35,36,37]. However, most of these studies need validation before clinical implementation.

The main advantage of saliva over blood sampling is the non-invasive nature of collection, which is particularly interesting for studies in children. Saliva sampling also facilitates and eases the collection of multiple subsequent samples for disease monitoring and longitudinal study designs. Collection and handling of saliva samples is relatively easy, does not require trained personnel and compared to blood, saliva samples cannot clot. In addition, saliva is safer compared to blood regarding the risk of transmission of viruses, such as HIV [33].

Although saliva seems to be the ideal source for measuring biomarkers, there are many variables regarding the collection and handling of samples [38]. Moreover, the composition and protein concentrations in saliva are influenced by many factors such as age, gender, hydration status, flow rate, time of sampling and diet [39].

In conclusion, saliva collection is simple and non-invasive and offers great opportunities. However, standardization of the methods for collection and handling of saliva samples is required before introduction in daily practice.

## 4. Conclusions

In recent years, biomarkers have been a growing field of study in medicine. Technical advancements now enable the measurement of many biomarkers in small volumes and have been illustrated to be extremely useful in medicine. The application of biomarkers in AD offers great opportunities, both in daily clinical practice as well as for research purposes.

Biomarkers offer reliable and objective outcome measures. Although serum TARC levels provide an excellent adjunct to daily practice and clinical trials, there are limitations to the use of a single biomarker in a complex disease such as AD. We are convinced that a panel of biomarkers will replace traditional outcome measurements in the near future. This will result in clinical trials being more “comparable”, which will prove to be essential with the introduction of biologicals in the treatment of AD.

The heterogeneous character of the disease and lack of clinical stratifiers makes AD highly suitable for a biomarker based stratification. A panel of biomarkers can assess multiple molecular entities, and will be more suitable for assessing disease severity in AD compared to an individual biomarker. Biomarker stratification will identify subgroups of patients that will respond to treatment and enable tailoring of drugs to the biological signature of individual patients, accelerating the translation of new medicine from bench to bedside and can revolutionize AD therapy.

Alternative sources for biomarkers such as saliva and capillary blood can increase the user friendliness of biomarkers in AD. Moreover, it will increase our understanding of the disease as they allow early sampling, for example during an exacerbation. Both DBS and saliva offer simple, non-invasive and cost effective alternatives to venipuncture [23,40]. The patients can complete collection procedures themselves and handling procedures are relatively simple. This offers great translational and clinical potential, not only for adults but also in pediatric populations. However, both methods need further validation.

In conclusion, biomarkers will play an increasingly important role in AD research and personalized medicine. The use of biomarkers will enhance the efficacy of AD treatment by facilitating the individualization of therapy targeting the patient’s specific biological signature and also by providing tools for predicting and monitoring of therapeutic response.
